# The Potential Associations of Pornography Use with Sexual Dysfunctions: An Integrative Literature Review of Observational Studies

**DOI:** 10.3390/jcm8070914

**Published:** 2019-06-26

**Authors:** Aleksandra Diana Dwulit, Piotr Rzymski

**Affiliations:** Department of Environmental Medicine, Poznan University of Medical Sciences, 60-806 Poznan, Poland

**Keywords:** pornography, sexual dysfunction, erectile dysfunction, delayed ejaculation, sexual desire, sexual satisfaction

## Abstract

This paper reviews the associations between pornography use and sexual dysfunction based on evidence from observational studies. The existing data in this regard mostly derive from cross-sectional investigations and case reports. There is little if no evidence that pornography use may induce delayed ejaculation and erectile dysfunction, although longitudinal studies that control for confounding variables are required for a full assessment. The associations between pornography use and sexual desire may differ between women and men although the existing data is contradictory and causal relationships cannot be established. The strongest evidence is available for the relation of pornography use with decreased sexual satisfaction, although the results of prospective studies are inconsistent. The paper outlines future research prospects beneficial in understanding the nature of associations between pornography use and sexual dysfunctions in men and women.

## 1. Introduction

The existing literature provides a number of varying descriptions of the term pornography. According to the Final Report of the Attorney General’s Commission on Pornography, it can be defined as any material that is predominantly sexually explicit and intended primarily for the purpose of sexual arousal [1]. Currently, pornography represents an important economic venture [2,3]. Its greatest development has occurred along with the emergence of computer technologies and the expansion of the Internet [4,5]. Due to a high sense of anonymity and almost unrestricted access, the Internet has become the most important medium of dissemination of pornographic content (known as online pornography), particularly in the form of images and videos [6,7]. The ease, arousal strength, and diversity with which pornography can be reached online indicates that it may operate as a supernormal stimulus [8].

**Figure 1:**
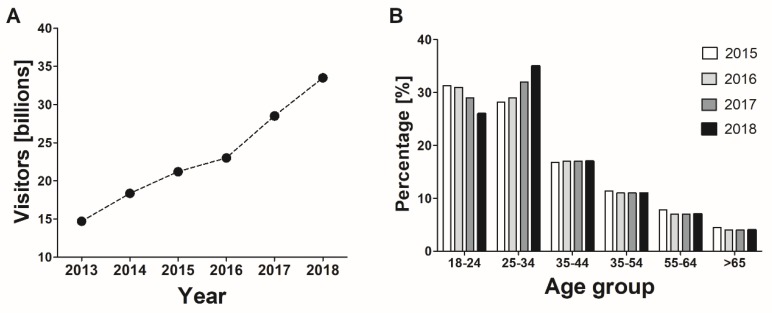
Statistics of pornography use in the period of 2013–2018 according to data shared by Pornhub: (**a**) annual number of visitors to Pornhub, (**b**) visitors to Pornhub by age.

According to various epidemiological studies, a relatively large number of adults have been exposed to pornography [9,10,11,12]. Recent representative surveys demonstrate that in developed countries with unrestricted Internet access, such as the United States and Australia, the majority of men (64–70%) and approx. one quarter/third (23–33%) of women are using pornography [13,14]. However, the number of pornography users is also relatively high in developing countries—recent surveys have shown that over half of students in Ethiopia and Bangladesh have been exposed to it [11,15]. The extensive use of pornography is also supported by data provided by Pornhub, one of the largest online pornographic websites, which clearly indicate that it is primarily men that are associated with content of this type (74%), and that the number of visitors to pornographic sites is growing from year to year (Figure 1). Some men deal with pornography on a regular, daily basis [16]. At the same time, the percentage of women interested in using this type of content is growing [17]. The Pornhub service is usually visited by young people under the age 34 from the United States, the United Kingdom and India. An emerging and as yet not fully assessed issue is the unintentional contact from advertising or spam e-mail messages both of which may sometimes be difficult to avoid [18].

Although interest in pornographic content can be partially considered as a natural element in the development of sexual experience in young people, the multiplicity and diversity of available online pornographic materials as well as the difficulty of restricting access to them lead to a question on the potential effects of pornography consumption. There is a steady increase in number of studies addressing the prevalence, patterns, outcomes, and various other aspects related to pornography use as clearly indicated by a systematic search of English language papers indexed in the PubMed/Medline database—a key term “pornography” yields 142 papers published in the period 1980–1989, 238 papers in 1990–1999, 524 papers in 2000–2009, and as many as 949 papers in 2010–2018. However, despite a continuous interest in the study of various aspects of pornography use, there are number of unresolved issues regarding the nature and magnitude of these effects. For example, some investigations demonstrate that pornography may fit into the addiction framework via mechanisms similar to chemical compounds [19,20] although controversies in this regard exist [21,22,23,24]. An addiction to pornography is not recognized in the DSM-5 and ICD-11 classifications (although the latter specifies a diagnostic category of Compulsive Sexual Behavior Disorder under impulse control disorders that may be used to diagnose problematic pornography use), various studies refer to it rather as “self-perceived pornography addiction” [12,16,25,26,27], and some alternative models based on moral incongruence, compulsivity, or impulsivity were also proposed to describe problematic pornography use [21,28,29]. Whether pornography may be associated with changes in sexual function is also a subject open to wide discussion. However, there are number of recognized risk factors for sexual dysfunction encompassing medical conditions, substance abuse, medication use, as well as cultural and social factors [30] which are difficult to address in studies focusing solely on pornography use. In the general population, the most frequently identified sexual dysfunctions include premature ejaculation and erectile dysfunction in men and desire and arousal dysfunction in women [31], and a number of studies have aimed to evaluate the potential associations between the occurrence of these effects and pornography use. At the same time, the potential effects of pornography use are the subject of a number of nonacademic discussions, and some publicly expressed opinions in this regard appear to be politically and ideologically driven. All in all, this creates a need to critically assess the existing evidence, outline study limitations and shortcomings, and highlight the future research prospects in the field of pornography use and its associations with sexual function.

The aim of this paper was to review the cross-sectional and longitudinal studies as well as case reports on potential associations between the use of pornography and sexual dysfunctions, namely erectile dysfunction, delayed ejaculation, and decrease in sexual desire and sexual satisfaction. These conditions are among the most often identified sexual dysfunctions in men and women [30,31,32]. Both quantitative (addressing the frequency of use) and qualitative (addressing the patterns of use) research was taken into account as these two approaches complement each other in understanding the complex nature of factors associated with pornography [33,34]. For this purpose, a systematic search for original research published since 2000 in peer-reviewed journals was performed using the PubMed/Medline and Scopus database, and by hand-searching reference lists from identified papers. The limitations of the conducted studies and future research prospects are also outlined.

## 2. Delayed Ejaculation

Delayed ejaculation describes a sexual dysfunction occurring in men, manifested by prolonged time required to ejaculate or complete inability to achieve it. Due to the complexity of psychosexual and psychosocial factors that contribute to its pathogenesis, there are no universal methods of treatment [35]. Its potential causes include, among many, frequent masturbation and the occurrence of significant discrepancies between real sexual intercourse with a partner and sexual fantasy preferred during masturbation [35,36]. Both masturbation and sexual fantasy are often associated with pornography use thus its potential relationship with the onset of delayed ejaculation is hypothetically plausible. A systematic search with key terms “pornography and ejaculation” and “pornography and delayed ejaculation” identified five original papers, including three cross-sectional studies and two case studies.

The first study to address the potential impact of pornography use on ejaculatory dysfunction was conducted on a group of 115 hypersexual, predominantly heterosexual men (mean age 41 years, range 19–76 years) [37]. As reported, a relatively significant percentage of subjects (23.5%; *n* = 27) masturbated chronically (at least 1 h/day or >7 h/week), usually while viewing pornography. In comparison with other subjects, this particular group was characterized by a higher anxiety level and was less likely to establish partner relationships or to persevere in them, even if they were established. These subjects frequently (19/27; 71%) reported some sexual dysfunctions with delayed ejaculation being reported the most often (in over 30% of cases). There are, however, a number of limitations to this study in the context of understanding the potential role of pornography in the occurrence of delayed ejaculation: (1) it only included hypersexual male subjects who represent a group that generally often masturbates and views pornography [38], and it remains unknown how these findings may be representative of the general population; (2) the onset of delayed ejaculation may result exclusively from the frequent masturbation or subjects with delayed ejaculation may tend to masturbate more often—in both cases, pornography use may remain unrelated; (3) it was unestablished whether the pornography use in hypersexual subjects facing delayed ejaculation preceded problems with this sexual dysfunction, therefore its role as a causative factor in delayed ejaculation cannot be established.

Two other cross-sectional studies involving young subjects do not support the potential existence of a relationship between pornography use and delayed ejaculation. The first of them surveyed Italian students attending their final year of high school (*n* = 1492; aged 18–19 years) who frequently admitted to using pornography (78%, including 8% using it on daily basis) and observed that ejaculatory issues were reported in 1% of surveyed, regardless of the frequency of pornography consumption [39]. In the second study, two large-scale samples of heterosexual men (aged 18–40 years) from three European countries, Croatia, Norway, and Portugal (*n* = 3948), were analyzed and, as demonstrated using multivariate logistic regression, no significant association between delayed ejaculation and pornography was detected [40].

In addition to cross-sectional studies, Park et al. [41] and Blair [36] reported cases in which delayed ejaculation appeared in some way to be related to pornography use. The former report described a case of a 20-year-old man with no chronic or mental disorder who used pornography for a long duration at a high frequency (1–2 times/day), gradually reaching for content that deviated progressively further from the standard. He also admitted to using an artificial vagina that supposedly allowed him to reach orgasm much faster. He self-reported the difficulty in maintaining an erection and ejaculating during masturbation and sexual intercourse, which contributed to disturbances in partner relations with his fiancée. As the authors emphasize, despite the fact that the man felt a physical and mental attraction to his partner, he preferred to use a more stimulating erotic toy (artificial vagina). The authors suggest that excessive pornography use could trigger changes in the nervous pathways responsible for sexual desire and erection, as well as changes in the functioning of the reward system, and subsequently caused delayed ejaculation [41]. These suggestions, however, remain purely speculative as no evidence to justify them was provided. As found, the delayed ejaculation was fully resolved after cessation of online pornography use and the quality of partner relationship was improved. However, the use of the artificial vagina was simultaneously discontinued. It therefore remains unestablished whether the delayed ejaculation was in any way related to the use of pornography, the artificial vagina, or both.

The case reported by Blair [36] included a 19-year-old male who could not achieve ejaculation during sexual penetration. The man started using pornographic content at the age of 12; a year later, he used it regularly, and at the age of 15 he began to reach for more and more thematic content (depicting the so-called bondage and acts of domination). Cessation of pornography and advice to avoid masturbation using a firm grip and switch to a more gentle style of penile stimulation were reported to be an effective therapy enabling the subject to achieve orgasm during an intercourse [36]. Therefore, this case also cannot be used as sole evidence for pornography-induced ejaculation impairment as it could just as well result from penile desensitization, a consequence of frequent masturbation. Some studies have reported that masturbation frequency and style, particularly the so-called “idiosyncratic” pattern that due to speed, pressure, and duration is difficult to be replicated by a partner, may be a predisposition for retarded ejaculation [42,43,44]. Therefore, the extent to which pornography use may contribute to such phenomenon remains unclear.

In summary, there is currently little evidence that an association between pornography use and delayed ejaculation exists and no indication that pornography use can be a cause of this sexual dysfunction. However, the assessment in this regard is only based on cross-sectional studies and case reports. Future research, particularly more extensive cohort studies and case-control observations, is therefore required.

## 3. Erectile Dysfunction

Erectile dysfunction is defined as a chronic inability to maintain an erection which prevents the introduction of the penis into the vagina. Its most common causes include age, diabetes, depression, cardiovascular and neurological diseases, selected psychogenic factors (including stress and abuse of psychoactive substances), and using selected pharmaceuticals [45]. Considering that some studies indicated a significant correlation between hypersexuality and problems with erectile function [46], it is plausible that some association in this respect may also exist for pornography use. A systematic search with key terms “pornography and erectile dysfunction”, “pornography and erectile function”, and “pornography and erection” identified a total of seven papers overall encompassing two case reports [41], six cross-sectional studies [28,39,40,47,48,49] and one longitudinal study [28].

Two interesting cases were presented by Park et al. [41]. In the first, a 40-year-old man with difficulty in maintaining an erection and achieving orgasm was described. During the period preceding the study he had intensively undertaken masturbation associated with the frequent use of online pornography, which was reported to be associated with an increasing amount of time required to achieve orgasm. He had also begun to view his wife as becoming gradually less sexually attractive. His physical parameters (including state of genitals) were in good condition. The patient was advised that his dysfunctions could have arisen from increased sexual stimulation, frequent masturbation, and change in the stimulation threshold due to exposure to strong pornographic content. The man, however, was unable to refrain from masturbation and watching pornography and did not initiate the treatment [41]. Another case described by the same authors concerns a 24-year-old man who was abusing alcohol and antidepressants, and had attempted suicide. He also reported to using online pornography at a frequency estimated at 5 h daily during the 6 months preceding the treatment. He experienced a weakened sexual interest in his wife, which was manifested by his inability to maintain an erection and preference to watching pornography, during which he experienced no erection problems. After discontinuing the use of pornography, according to the therapist’s recommendation, his erectile dysfunction disappeared [41]. Both of these cases are complicated with confounding variables and no casual relation between pornography use and erectile dysfunction can be seen. In the first, it is not possible to separate the potential effects of frequent pornography use and excessive masturbation, although one should note that these two phenomena can often be highly correlated in men [50]. The second case is complicated by psychiatric history (use of antidepressants and suicide attempt) as well as by the reported alcohol abuse which itself is a common cause of sexual dysfunctions such as erectile retardation [51].

As found in a pilot observational study conducted in 2006 on a small group of young adult men (*n* = 25; mean age 29 years), nearly half of them (*n* = 12) showed no signs of sexual arousal, including erections while watching an erotic film (penile rigidity < 5%; and 0% in eight subjects) [47]. These observations were initially associated with a potentially high level of exposure to pornographic content, lowering the responsiveness to sexual stimuli associated with the presentation of sex in a more standard edition (vanilla sex). In the second stage of the study, a larger number of men were recruited (*n* = 80) and exposed to longer and more diverse erotic films. Nineteen percent of them (*n* = 15) failed to respond sexually. It appeared that the risk of sexual dysfunction increased along with the number of pornographic films that had been viewed by the respondents during the previous year [47]. Another study of a larger range was conducted in 2016 on a group of 434 men (mean age 29.5 years, range 18–72). Using the International Index of Erectile Function questionnaire, the ability to achieve an erection and orgasm, the degree of sexual desire, satisfaction with sexual intercourse, and general sexual satisfaction were evaluated in 276 subjects who had had sexual intercourse during the last month. The study concluded that problematic online sexual behavior (defined as compulsive, persistent, uncontrolled use of pornographic content) was a significant predictor of a low level of erection [49].

In turn, the study surveying Italian high school students (*n* = 1429; age 18–19 years) did not show that erection problems were more frequently admitted by teenagers watching pornography, regardless of the self-reported frequency of its use [39]. A cross-sectional study conducted in two-large scale samples on heterosexual men (aged 18–40 years): the first in 2011 on Croatian, Norwegian, and Portuguese heterosexual men (*n* = 2727) and the second in 2014 on another sample of Croatian men (*n* = 1211) identified a positive relationship between pornography use and erectile dysfunction in the first subset of individuals from Croatia although the effect was small and not confirmed in other groups [40]. Another study reported that instead of erectile dysfunction, pornography use in 280 heterosexual men (mean age 23 years) was positively correlated with sexual arousal which was self-reported when watching visual stimuli in the laboratory [48]. Furthermore, subjects indicating higher pornography consumption also reported a greater desire for solo and partnered sexual behaviors. However, this study had a number of limitations: a high number of monogamous individuals (which may be more sexually exploratory, particularly if young), a rather limited frequency of pornography use in the studied group (individuals were divided into three groups using pornography 0, 1–2, and >2 h per week but the maximum frequency remained unreported), and an unknown period of pornography use in the investigated individuals prior to the study.

The most recent study performed by Grubbs and Gola [28] reported a positive association between self-reported erectile dysfunction and self-reported problematic pornography use but not mere pornography use in a cross-sectional sample of 147 undergraduate men (mean age 20 years) in the United States as well as in a sample of 433 men (mean age 33 years) matched to the demographic norms of this country. The one-year, four-wave longitudinal study that was based on these two samples, completed across all four time points by 117 participants, and with two point-data collected for 278 subjects, also found that baseline pornography use and problematic pornography use was positively associated with prospective erectile dysfunction. However, latent growth modelling indicated that no baseline variables served as predictors of the trajectory of erectile functioning over time. Although these results support the existence of an association between erectile dysfunction and problematic pornography use, they fail to show a causal relationship. It is thus plausible that men with erectile dysfunction may tend to use more pornography, including patterns they self-perceive as problematic [28].

As yet, there is little or no evidence on a causal relationship between erectile dysfunction and frequency of pornography use. It cannot be ruled out that subjects with erectile dysfunction may be more prone to using pornography more frequently. One should note that cross-sectional and longitudinal studies performed so far are solely based on self-reported data introducing a significant limitation. Some research clearly indicates that the prevalence of self-reporting of erectile dysfunction may vary considerably from the prevalence identified by objective methods such as the International Index of Erectile Function to the extent that the former might be unreliable in assessing the real presence of this sexual dysfunction [52]. There is a need for further longitudinal exploration of associations between erectile dysfunction and pornography use that would include individuals of different age and with various baseline pornography use and employ a diverse methodology encompassing physiological measures and partner reports.

## 4. Changes in Sexual Desire

From the perspective of biological sciences, the term libido is used to describe sexual desire, a trait controlled by central nervous system associated with the sexual drive and wish to engage in sexual activities [53]. As highlighted, it should not be mistaken for sexual arousal which manifests itself physiologically and may not always be positively correlated with sexual desire [54]. This said, it can be hypothesized whether pornography use increases or decreases libido, and if frequency and duration of pornography consumption may modify such responses. One can also consider different responses in males and females due to varying sex roles and sexually differentiated neural activity in response to sexual stimuli [55]. To explore it, a systematic search for original studies was performed with the key terms “pornography and libido” and “pornography and sexual desire”. A total of five papers associated with this subject were identified and included cross-sectional studies [39,40,50,56,57].

Carvalheira, Træen, & Stulhofer [50] analyzed the relationship between masturbation and the use of pornography and sexual desire in a group of European heterosexual men (mean age 40 years, range 21–73) who had reported a problem of reduced sexual desire (*n* = 596). As found, more than half of the studied subjects who had experienced a significant decrease in libido within six months before the examination were involved with pornographic materials at least once a week. The study further found that frequency of masturbation and pornography use are strongly correlated in men with decreased sexual desire. One should note that the cross-sectional nature of this study does not allow any causation between pornography consumption and decreased libido to be established, and that interpretation of the obtained data is also limited by the lack of a control group constituted by men with no sexual dysfunctions. Although it is generally an interesting or even counterintuitive observation that men with an impaired libido may watch more pornography and masturbate often, it is important to highlight that men with lower sexual desire (contrary to women with lower libido) tend to increase the frequency of masturbation in a manner unrelated to pornography consumption [58,59]. Considering the high accessibility of online pornography, it is no surprise that men who tend to masturbate often will also constitute a group using it as sexual stimuli.

Cross-sectional observations in Italian students attending the last year of high school (*n* = 1492, aged 18–19 years) indicated that as many as 78% of them admitted to using pornographic content, with 8% indicating doing so on a daily basis. A decrease in sexual desire was reported by 10% of pornography users, and appeared to increase with the frequency of consumption: among students exposed at least once a week, it accounted for 16%, while in the case of those exposed less often it was 6%; the nonusers did not report it at all [39].

The findings of Carvalheira, Træen, & Stulhofer [50] and Pizzol, Beroldo, & Foresta [39] were not confirmed in a large study encompassing large-scale samples of heterosexual men (aged 18–40 years) from Croatia, Norway, and Portugal (*n* = 3948) and applying multivariate logistic regression [40]. In turn, a study on women (*n* = 754; aged 18 = 76 years) reported that those involved in a long-term relationship that use pornography more frequently may reveal increased sexual desire towards their partners and report a higher desire for sexual variety [56]. This is a relatively important finding indicating the potential difference in patterns of pornography use between men and women, although one should note that the cross-sectional nature of the study does not imply causation. It remains to be explored whether a pornography-induced increase in libido exists in women or women with higher sexual desire are also more open to watching pornography more frequently. Moreover, the potential role of sexual partner (in terms of sexual desire and satisfaction) and satisfaction from a relationship may represent important factors for inclusion in multivariate analyses conducted in the future. Interestingly, a recent cross-sectional survey of 240 committed heterosexual couples (mean age of males and females 35 and 33 years, respectively) confirmed the positive correlation of pornography use by women with their sexual desire but also found a similar but weaker relation in men [57].

Neurobiological research indicates that the potentially negative effect of long-term pornography use on sexual desire may result from changes in the responsiveness of the reward system to sexual stimuli, preferentially more active as a result of stimuli associated with pornography than with real sexual intercourse [60,61]. However, observational studies do not provide consistent data to support the hypothesis that use of pornography is a causative factor for a decrease in sexual desire and rather provide a contradictory observation as regards the existence and direction of correlations between pornography use and libido. These contradictions may potentially arise from the complex nature of sexual desire in both men and women, which is influenced by a number of biological, psychological, relational, sexual and cultural factors [62,63]. Considering that some studies have reported that subjects with higher sexual boredom and lower libido may tend to masturbate more frequently [50], it is important to elucidate the role the pornography use and pornography-associated masturbation may play in fulfilling the need for sexual gratification. Further cross-sectional studies as well as prospective investigations that control for these factors are greatly required to draw some final conclusions on the relation of pornography use and level of sexual desire.

## 5. Changes in Sexual Satisfaction

It could be hypothesized that the frequent exposure to pornography can potentially impact sexual satisfaction. The potential reasons for its decrease may include: (1) a comparison of real partners to idealized acting roles in pornographic films [64,65], (2) disappointment when the actual partner is not interested in recreating the scenes observed in pornographic material, (3) disappointment due to the inability to obtain such a broad spectrum of sexual novelties, with a real partner as presented in pornographic material [66,67] and (4) contact with pornography chosen instead of sexual intercourse with a real partner [68,69].

On the other hand, one could also hypothesize that in some cases, use of pornography may increase sexual satisfaction by providing inspiration for real sex. However, the magnitude of these effects may differ between men and women, and may also be potentially modified by frequency and time of pornography use, as well as type of pornography consumed. Moreover, it may also be hypothesized that shared pornography use in couples may have a positive impact on sexual satisfaction as it could stimulate partners for more sexual exploration during real intercourse [70].

A systematic search with the key term “pornography and sexual satisfaction” identified a total of 23 papers reporting observational studies among which 20 cross-sectional surveys (Table 1) and four prospective investigations were reported [65,71,72,73].

As found, the associations of pornography use on sexual satisfaction may differ across gender (Table 1). In general, its decrease was more often observed in men than women. Moreover, the frequency of pornography use may also be differentially associated with sexual satisfaction in both genders—in men, its decrease was already reported at a rate of use estimated at a few times per year while in women at a frequency of once a month [74]. As demonstrated in both women and men, age of first exposure may also be associated with decrease in sexual satisfaction, with a two-fold increase in the odds if such exposure occurred ≤ 12 years in reference to individuals exposed for the first time >16 years. Nonreligious individuals and those in a relationship were also found to reveal weaker associations between pornography use and sexual satisfaction [74,75]. Interestingly, one study reported that, in men, the negative association between pornography use and sexual satisfaction appeared to diminish once masturbation frequency was controlled [76]. However, one should note that pornography consumption and masturbation are usually highly associated in men [50]. Altogether, it highlights that the context of pornography use may highly moderate the nature of associated effects and should be taken into account in further assessments. As recently indicated, length of relationship was negatively associated with pornography use in women, thus mitigating its effects on sexual satisfaction [77]. In turn, in newly married couples, pornography use was demonstrated to be negatively correlated with sexual satisfaction [73]. One study demonstrated that the association between pornography use and sexual satisfaction may be differentiated according to the attachment styles of the studied subjects: with no association found in secure individuals (neither anxious nor avoidant), a negative association among preoccupied (high anxiety but low avoidance) and dismissing (low avoidance and high anxiety) subjects and a positive one among fearful persons (simultaneously highly anxious and avoiding) [78]. This puts an association between pornography use and sexual satisfaction in a wider psychological context in which it may arise from early interactions with caregivers that, via internalization of operative cognitive models, guide behavior and cognition, in relation with sexuality in adulthood. This is particularly interesting in the light of the research of Szymanski and Stewart-Richardson [79] who demonstrated that frequency of pornography use as well as problematic pornography use in heterosexual men is related to more avoidant and more anxious attachment styles. The authors hypothesize that these men use pornography as it allows them to experience emotional and/or sexual gratification without having to risk interpersonal rejection or intimacy [79]. Altogether, these findings suggest that the nature of associations between pornography use and sexual satisfaction may depend on various variables encompassing gender, relationship status, cultural/religious factors, and psychological background, and this, in addition to quantitative data, should be taken into account in future studies.

Overall, it appears that individuals, particularly men, who use pornography more often also tend to report lower satisfaction with their sex life. The limitations of cross-sectional studies do not allow us to distinguish whether pornography induces a decrease in sexual satisfaction or whether low sexual satisfaction predicts more frequent pornography consumption, or both. The first longitudinal, three-wave (six months between waves) panel study in this regard conducted on a population of Dutch adolescents (*n* = 1052; aged 13–20 years) revealed that pornography use consistently reduced sexual satisfaction but also that low sexual satisfaction led to increase in pornography use [65]. This highlights that these bidirectional relationships must be taken into account, and that other factors contributing to lower sexual satisfaction (that may potentially include sexual or psychosocial dysfunctions) should be addressed to fully elucidate the reasons for pornography use. Gender was not demonstrated to be a moderator of observed effects in this study. However, another four-wave panel study (six months between waves) which was also conducted on a sample of Dutch adolescents (*n* = 1132; aged 11–18 years) indicated that more frequent pornography use at baseline predicted less sexual satisfaction at the last study point in males, while in females, sexual satisfaction was negatively associated with an increase in pornography use [71]. The findings by Peter & Valkenburg [65] and Doornwaard et al. [71] were not replicated in the other, three-wave longitudinal study (one year between waves) that surveyed 190 newly married heterosexual couples [73]. As demonstrated, the frequency of pornography use in women and men failed to predict changes in sexual satisfaction and pre-existing sexual satisfaction did not predict changes in pornography consumption. More recently, a longitudinal six-wave study (six months between waves) of females (*n* = 775) and males (*n* = 514) aged 15–18 years, also found no significant association between frequency of pornography use and sexual satisfaction, regardless of gender [72]. Further prospective studies will be necessary before any definite conclusions can be drawn.

Importantly, the majority of cross-sectional studies summarized in Table 1 only assessed individual pornography use. As shown by Willoughby & Leonhardt [57], shared viewing of pornography in heterosexual couples is correlated with higher sexual satisfaction. As demonstrated, women using pornography may more often experience guilt, disgust, and embarrassment [90], which are rather not experienced when this use is shared with their partners—such a scenario may promote positive sexual interactions in couples. One should note, however, that the findings of Willoughby & Leonhardt [57] are derived from a cross-sectional study and no causality can be established. It can be hypothesized that shared pornography use increases sexual satisfaction in partners or that partners experiencing higher sexual satisfaction may tend to view pornography together more often.

Additionally, interesting associations between pornography use and a partner’s sexual satisfaction have been reported. For example, Yucel and Gassanov [91] who surveyed 433 heterosexual married couples observed that a husband’s pornography consumption was negatively correlated with his wife’s sexual satisfaction while a wife’s pornography use was not associated with her husband’s satisfaction. In turn, the longitudinal observations made in 190 newlywed couples found that increased sexual satisfaction in men was a predictor of a decline in pornography viewing by their wives [73]. These bivariate associations suggest that gender patterns in pornography consumption in couple relationships may mutually affect sexual satisfaction and may be important to consider in future works on the effects of pornography use on the quality of sex life.

In conclusion, the accumulating evidence from cross-sectional studies supports the hypothesis that pornography use is associated with lower sexual satisfaction. However, the magnitude of this association appears to depend on a number of factors, including gender, relationship status, frequency, duration, and pattern of pornography use, and the age at which pornography use was initiated. One should also note that although much attention is paid to associations with lower sexual satisfaction, some studies not only report no associations of pornography consumption in this regard in large number of surveyed subjects but also indicate that some individuals experience an increase in sexual satisfaction. For example, in a recent cross-sectional study of Polish students who admit to current pornography use, respectively 68% and 7% associated its consumption with no effect and a beneficial effect on sexual satisfaction [12]. Moreover, studies in couples demonstrate that use of pornography may not necessarily be associated with less sexual satisfaction, and that in some cases, a positive correlation can be observed [57]. As already shown in relation to relationship quality, it is highly plausible that the nature of the association between pornography use and sexual satisfaction in individuals in romantic relationships may not only depend solely on the frequency of use but the context in which it is consumed, such as concordance or discrepancy in partners’ use, levels of acceptance of pornography use from both partners, known or hidden use, and individual or shared use [70,92]. Moreover, the longitudinal studies conducted so far have failed to fully confirm that pornography use is a causative factor in impaired sexual satisfaction. It remains to be explored whether the potential effect of pornography in this regard can be influenced by: (i) sexual orientation: the majority of studies have focused on heterosexual individuals while homo- and bisexual individuals may even be more frequent pornography users as preliminarily found in men [93], (ii) physical disabilities as they may also influence a baseline sexual satisfaction [94], and (iii) co-occurrence of other sexual dysfunctions, as some authors have indicated that pornography use may be a continuation of pre-existing compulsive sexual behaviors [64,95].

## 6. Future Research Prospects and Conclusions

Increasing access to the Internet has opened a completely new chapter for the pornographic industry, simultaneously increasing both the time and strength of exposure to pornographic content, and its potential effects on health. The studies conducted so far indicate a correlational relationship between pornography consumption and selected sexual dysfunctions with the strongest evidence for a decrease in sexual satisfaction. It should be noted that the vast majority of observations are based on cross-sectional studies or case reports and without future research based on extensive case-control and/or prospective cohort studies, causality cannot be comprehensively assessed. One should also note that assessment of pornography use in studies is mostly based on self-reporting and that objective confirmation of exposure is not possible. Moreover, the presence of sexual dysfunctions such as erectile dysfunction is also often self-reported and creates the risk of their being underestimated; thus, when possible, the use of validated tools is advised. There are number of recognized risk factors for sexual dysfunctions which need to be considered when evaluating the potential effects of pornography use in future studies. The frequency of pornography use may in turn be potentially modulated by various parameters such as gender, cultural/religious factors, relationship status, and psychological background. Further research on associations between pornography consumption and sexual dysfunction should also take these into account. Unlike the effect of psychoactive substances or binge eating, the potential effects of pornography use cannot be recreated using experimental animal models, while the scope of experimental research involving human volunteers is rather limited and can often only be used to assess short-term outcomes. This in turn highlights the need for more, well-designed observational, particularly prospective studies. To provide a broad insight into the potential associations of pornography use with sexual dysfunctions, it would be best for future studies to provide a definition of pornography, specify the type of pornographic content consumed by the studied subjects (e.g., violent, nonviolent, mainstream, and paraphilic), control for the frequency of masturbation, consider the sexual orientation of participants, whether they are in a relationship or not, and if they are what is their relationship satisfaction and whether they consume pornography individually or in a shared manner. The context in which pornography is consumed rather than the mere use may moderate the associated effects, and such context must be taken into account in further assessments. The complexity of factors influencing pornography use and modulating its associated effects, as well as the susceptibility of research models to methodological biases and difficulties in overcoming the limitations of studies strongly justify a need for further investigation on the associations between sexual functionality and pornography consumption, which is particularly important given the high rates of the latter.

## Figures and Tables

**Table 1:** Cross-sectional studies on association between pornography use and sexual satisfaction in women and men.

Study Type	Group	Age of Subjects Mean ± SD (range) years	Method	Observation	Reference
Online survey	373 heterosexual men	19 ± 2 (18–29)	Multidimensional Sexuality Questionnaire	Frequency of PU and PPU correlated with ↓ sexual satisfaction	[79]
Online survey	217 heterosexual couples	37 ± 11 ♂; 35 ± 10 ♀	Index of Sexual Satisfaction	Frequency of PU correlated with ↓ sexual satisfaction only in male	[80]
Online survey	650 men	(18–25)	Snell’s Index of Sexual Satisfaction	Earlier exposure to P correlated with ↓ sexual satisfaction	[81]
Online survey	326 heterosexual men; 456 heterosexual women	20 (18–30)	One-item question	Frequency of PU correlated with ↓ sexual satisfaction only in male	[82]
Online survey	221 women; 75 men; (97% heterosexual)	29 ± 9 (18–87)	Index of Sexual Satisfaction	No correlation between frequency of PU and sexual satisfaction	[83]
Online survey	1513 heterosexual adults	23 ± 8	Two-item question	Frequency of PU correlated with ↓ sexual satisfaction	[74]
Online survey	240 heterosexual couples	35 ± 9 (18–72) ♂; 33 ± 9 (18–60) ♀	Golombok Rust Inventory of Sexual Satisfaction	Couple PU correlated with ↑ sexual satisfactionUnknown individual use correlated with ↓ sexual satisfaction	[57]
Pen-and-paper survey	1501 randomly selected adults	50 ± 18 (17–98)	One-item question	Frequency of PU correlated with ↓ sexual satisfaction only in male	[75]
Online survey	565 women; 471 men	(18–55)	One-item question	Frequency of PU correlated with ↓ sexual satisfaction only in male	[77]
Online survey	894 heterosexual adults	30 ± 9	Two-item question	Frequency of PU correlated with ↓ sexual satisfaction with no gender differences	[84]
Online survey	596 women; 234 men	25 ± 8 (18–78)	Global Measure of Sexual Satisfaction	Frequency of PU correlated with ↓ sexual satisfaction, particularly lower scores were seen in compulsive users.	[85]
Online survey	587 women; 232 men	25 ± 8 (18–78)	Global Measure of Sexual Satisfaction	Frequency of PU correlated with ↓ sexual satisfaction in both gender	[86]
Online survey	1471 women; 1109 men	(18–60)	New Scale of Sexual Satisfaction	Frequency of PU correlated with ↓ sexual satisfaction in both gender	[87]
Pen-and-paper survey	190 newly married heterosexual couples	34 ♂; 31 ♀	Perceived Relationship Quality Components (PRQC) Inventory	Frequency of PU correlated with ↓ sexual satisfaction in both gender	[73]
Face-to-face interviews	2610 married adults	53 ± 14 (25–80)	One-item question	Frequency of PU correlated with ↓ sexual satisfaction in studied group	[88]
Online survey	433 heterosexual married couples	38 (22–59) ♂; 35 (20–44) ♀	One-item question	No correlation between frequency of PU and sexual satisfaction in husbands and wives	[79]
Pen-and-paper survey	326 heterosexual couples	38 ± 10 ♂; 36 ± 10 ♀	Golombok Rust Inventory of Sexual Satisfaction	Frequency of PU correlated with ↓ sexual satisfaction in both gender	[89]
Pen-and-paper survey	460 women; 130 men	24 ± 7 (18–64)	One-item question with Likert scale	PU correlated with ↓ sexual satisfaction. The association was differentiated by attachment styles: negative among anxious/avoidant subjects, positive among fearful individuals	[78]
Online survey	3004 women; 2079 men	22 ± 1 (18–26)	One-item question	Earlier age of exposure to P increased odds for ↓sexual satisfaction	[12]
Online survey	470 men	27 ± 11	Global Measure of Sexual Satisfaction	Frequency of PU correlated with ↓ sexual satisfaction	[76]
Online survey	378 men	47 ± 14	Global Measure of Sexual Satisfaction	Frequency of PU correlated with ↓ sexual satisfaction	[76]

P—pornography PU—pornography use; PPU—problematic pornography use; SD—standard deviation.
